# Modeling the structural relationships between trauma exposure with substance use tendency, depression symptoms, and suicidal thoughts in individuals with earthquake trauma experience: the mediatory role of peritraumatic dissociation and experiential avoidance

**DOI:** 10.1186/s12888-024-05595-5

**Published:** 2024-03-01

**Authors:** Farshad Ahmadi, Mohammad Ali Goodarzi, Mohammad Reza Taghavi, Mahdi Imani

**Affiliations:** Department of Clinical Psychology, Faculty of Educational Sciences and Psychology, University of Shiraz, Shiraz, Iran

**Keywords:** Earthquake, Substance use, Depression symptoms, Suicidal thoughts, Peritraumatic dissociation, Experiential avoidance

## Abstract

**Background:**

Despite the fact that studies indicate that earthquake trauma is associated with numerous psychological consequences, the mediating mechanisms leading to these outcomes have not been well-studied. Therefore, this study investigates the relationship between trauma exposure with substance use tendency, depression, and suicidal thoughts, with the mediating role of peritraumatic dissociation and experiential avoidance.

**Methods:**

The descriptive-correlational approach was employed in this study. The participants were people who had experienced the Kermanshah earthquake in 2017. A total of 324 people were selected by convenient sampling method. The Traumatic Exposure Severity Scale, the Peritraumatic Dissociative Experiences Questionnaire, the Acceptance and Action Questionnaire, the Iranian Addiction Potential Scale, Beck’s Depression Inventory [BDI-II], and Beck’s Suicidal Thoughts Scale were used to collect data. The gathered data was analyzed‌ using structural equation modeling in ‌SPSS Ver. 24 and LISREL Ver. 24.

**Results:**

The study findings indicated that the intensity of the trauma exposure is directly and significantly associated with depression symptoms, peritraumatic dissociation, and experiential avoidance. The severity of exposure to trauma had a significant indirect effect on the tendency to use substances through experiential avoidance. This is while the severity of the trauma experience did not directly correlate with substance use and suicidal thoughts. In addition, peritraumatic dissociation did not act as a mediator in the relationship between the severity of trauma exposure with substance use, depression, and suicidal thoughts.

**Conclusions:**

The severity of exposure to the earthquake was associated with symptoms of depression and these findings indicate the importance of experiential avoidance in predicting the tendency to use drugs. Hence, it is essential to design and implement psychological interventions that target experiential avoidance to prevent drug use tendencies and to establish policies that lower depression symptoms following natural disasters.

## Background

Earthquakes are one of the most common natural disasters in the world [1]. Situated on the Alpine-Himalayan Seismic Belt [2], Iran is prone to these natural disasters. On November 12, 2017, an earthquake with a magnitude of 7.3 occurred in Western Iran near the Iran-Iraq border in the province of Kermanshah. The earthquake left 630 people dead, 9,388 wounded, and 70,000 homeless [3]. Many studies have shown that earthquakes have caused extensive material, human, and psychological damage in different regions of the world [4] particularly in Kermanshah [2, 3, 5]. Most studies are mainly focused on the development of post-earthquake PTSD. This is while other common psychological reactions to earthquake trauma experience can include substance use [4], depression [6], and suicidal thoughts [7]. Therefore, it is essential to understand the significant mediating mechanisms that influence the relationship between earthquakes and psychological consequences.

To comprehend the significant mediating mechanisms, one can rely on some theoretical and research foundations. Studies have shown that psychological disorders, including PTSD, can arise as a result of pre-existing psychological vulnerabilities and psychological distress following an earthquake [3, 8]. The evidence also emphasizes the importance of various types of psychological distress– dissociation– during and after the earthquake, in the formation of psychological outcomes [8, 9].

One of the key mediating mechanisms between psychological problems and earthquakes is dissociation [8, 9]. Many researchers consider dissociation as a common response to trauma [10, 11]. The dissociation model underlines that traumatized individuals can manage their distress by separating their awareness from their experience. The model suggests that peritraumatic dissociative responses (e.g., dissociative amnesia, emotional numbing, and depersonalization) indicate a strategy to reduce awareness of aversive emotions [12]. Dissociative experiences are a crucial aspect of the dissociative response to trauma, occurring during or immediately after a traumatic event, also called peritraumatic dissociation (PD) [13]. This component is a significant risk factor for PTSD and other psychological symptoms [14]. It is believed that PD occurs due to severe traumatic distress, including fear, helplessness, or trauma-induced horror [15] and it may function to regulate aversive peritraumatic affects [11]. Given the main function of dissociation in regulating specific aspects of trauma, such as aversive affect, it is not surprising to claim that dissociation is an essential factor in increasing the risk for depression [16, 17], suicidal thoughts [18–20], and the tendency to use drugs [21].

Drug consumers ‌may use drugs or alcohol to manage aversive affects‌ associated with traumatic dissociation. Sometimes, the substance user intends to access traumatic memories. Research also indicates higher levels of dissociation in drug users [21]. Dissociation also plays a vital role in the psychopathology of depression [22]. From a clinical point of view, depression can be explained by considering dissociation as a way of coexisting with despair. According to Pettorruso et al. [22], dissociation is a mechanism used by individuals experiencing depression to deal with stressful mental processes, tolerate the lack of positive future prospects, and emotional pain and distress. Additionally, emotional dysregulation, disinterest in social interactions, psychological distancing, excessive engagement in imagination, disturbing thoughts and memories, personality disintegration, and reality disintegration are among the signs related to dissociation that can significantly contribute to explaining depression [23, 24]. In the context of dissociation and suicidal thoughts, it can also be argued that chronic stress can lead todissociative experiences and severe vulnerability to stress, which can enhance suicidal behavior and thoughts when faced with intolerable stress, helplessness, and hopelessness [19]. In addition, dissociation can be understood as a form of detachment and can include depersonalization, derealization, amnesia, fugue states, and identity disorders [25]. Therefore, dissociation can involve a disconnection from the body that can reduce the fear and pain associated with harming the body that can make suicide attempts possible [18].

Aside from the current research, some argue that the association between the earthquake and its psychological consequences and the relationship between dissociation and psychological symptoms remain equivocal, suggesting the inclusion of some dissociative constructs, such as experiential avoidance for a better understanding of psychological phenomena related to trauma and dissociation [12, 26].

Experiential avoidance is another mediating mechanism that explains the impact of trauma on psychological consequences. An increasing number of theories and studies show that avoidance plays a significant role in the formation and maintenance of psychological symptoms among trauma survivors [27, 28]. Since PD can serve in forming avoidance or altering the aversive aspects of a traumatic event, it has been suggested that PD may be a form of experiential avoidance (EA) [27]. Experiential avoidance is a form of unwillingness to make contact with personal experiences (physical sensitivities, ‌feelings,‌ thoughts, memories‌, and ‌behavioral states) and an attempt to avoid those agonizing experiences ‌or events that cause the recollection of these experiences‌ [27]. EA controls or minimizes the impact of aversive experiences, resulting in short-term relief. As a result of this, behavior is negatively reinforced [29]. Hays et al. [27] state that trying to distance one self from internal experiences can lead to psychological and behavioral issues, such as substance use, depression, suicidal thoughts, and aggressive behaviors‌. Thus, the psychological and behavioral problems of trauma survivors may be a byproduct of the EA process.

Finally, it is important to review the literature related to the study variables. Marx, & Sloan [28] found that while EA and PD were initially ‌‌important‌ predictors of PTSD symptoms, after 4 to 8 weeks, only EA was associated with PTSD symptoms. The findings of Kumpula et al. [30] demonstrated that both EA and PD exert distinct influences as risk factors for PTSS after an event that is potentially traumatic. Different studies report increased or decreased psychological problems after earthquakes [31, 32, 33]. The role of dissociation on substance use, depression, and suicidal thoughts [20, 21, 22] and the role of EA ‌in substance use, depression, and suicidal ideation‍ [27, 34, 35] have been investigated in some studies.

Despite extensive research and significant theoretical support, the relationship between trauma and some psychological consequences (including substance use, depression, and suicidal thoughts) is still unclear. Therefore, to gain a better understanding ofthe psychological consequences associated with earthquake trauma, it is necessary to incorporate some mediating mechanisms (such as PD and EA ). In addition, since Iran is located in one of the most earthquake-prone areas in the world, identifying the psychological ‌consequences of earthquakes and their risk factors is vital: Accordingly, the present study was conducted to explore the relationship between trauma exposure and substance use tendency, depression, and suicidal thoughts in individuals with earthquake trauma experience through the mediating role of PD and EA‌.

Hypotheses of the present study:

1. Exposure to trauma directly affects the tendency to use substances, suicidal thoughts, and develop depressive symptoms.
2. Peritraumatic dissociation mediates the relationship between trauma exposure with substance use tendencies, depressive symptoms, and suicidal ideation.
3. Experiential avoidance mediates the relationship between trauma exposure with substance use tendencies, depressive symptoms, and suicidal thoughts.

## Methods

### Participants

The participants were people who had experienced the Kermanshah earthquake (residents of two cities of Sarpol-e Zahab and Salas-e Babajani) in 2017. The sample size was determined based on Kline’s [36] suggestion, which proposes a minimum sample size of 300 in SEM. In this study, to reduce the error margin and improve the generalizability of the results, a total of 323 people were selected by convenience sampling from residents struck by the earthquake. The inclusion criteria are the following: those who, at the time of the earthquake on November 12, 2017, as well as during this study, lived in one of the cities in Kermanshah (Sarpol-e Zahab Sallas-e Babajani), those who were completely fluent in the official language, and aged between 18 and 50 years. The exclusion criteria are the following: incomplete questionnaires and individuals with a history of hospitalization for substance use, depression symptoms, and suicidal thoughts before the earthquake.

### Procedures

This descriptive-correlational study was conducted based on structural equation modeling (SEM).

In this study, the data collection phase from struck residents in the cities of Kermanshah started after obtaining a recommendation letter from the University of Shiraz, coordinating with the Health Department of the Kermanshah University of Medical Sciences, and obtaining the permits required. In this study, the data were collected personally by the researcher. For each subject, the tests were performed separately by the researcher in a quiet room, away from aversivevisual-auditory stimuli This research has an ethics approval certificate (IR.SUMS.REC.1400.813) issued by the Shiraz University of Medical Sciences. This study was performed in line with the principles of the Declaration of Helsinki. To implement the ethical considerations, the codes of conduct proposed by the American Psychiatric Association, including the principles of privacy and confidentiality, written consent, etc. debriefing on processes, objectives, and duration, potential losses and benefits of participation, and the permission to withdraw from the research at any stage, were taken into account.

### Measures

#### The traumatic exposure severity scale (TESS) (earthquake-specific)

The severity of exposure to earthquakes is measured using its Persian version. The primary version of this questionnaire comprises 24 items with five sub-scales [37]. The Persian version of this questionnaire was provided by Nobakht et al. [38] and was normalized on the Iranian sample. It consists of 21 items divided into four sub-scales, including being in need/damage to home, personal harm, harm to significant others, and exposure to the grotesque. In the Persian version, participants were asked to use a 5-point Likert scale, ranging from 1 (Not at All) to 5 (To a Great Extent), to indicate the extent to which each item is distressing to them. In TESS, the distress scale was calculated by summing up the scores ranging from 1 to 5. TESS has shown in both the original [37] and Persian [38] versions that have demonstrated sufficient validity and internal consistency.

#### The peritraumatic dissociative experiences questionnaire (PDEQ)

The Peritraumatic Dissociative Experiences Questionnaire (PDEQ) measures PD. It is used to evaluate dissociative experiences and reactions during and immediately after a traumatic event [3]. PDEQ is a self-report measure. It consists of ten items scored on a 5-point Likert scale ranging from 1 (Not True at All) to 5 (Very True). The Persian version of this scale was normalized by Nobakht et al. [38] and demonstrated good validity, internal consistency, and reliability.

#### The acceptance and action questionnaire (AAQ-II)

This questionnaire was developed by Bond et al. [39]. In addition, a 10-item version of the original questionnaire (AAQ- I) was developed by Hayes et al. [40]. Psychological flexibility and experiential avoidance are measured using this questionnaire. The items are rated on a 7-point Likert scale according to the level of agreement. Higher scores on a scale indicate lower psychological flexibility and higher EA. The findings indicate that this instrument demonstrates acceptable reliability, validity, and construct validity, with a mean alpha coefficient value of 0.84. The test-retest reliability at an interval of 3 to 12 months was 0.81 and 0.79, respectively [39]. Abasi et al. [41] also demonstrated the acceptable internal consistency and convergent validity of the Persian version of this questionnaire.

#### The Iranian addiction potential scale (IAPS)

This questionnaire was designed by Weed et al. [42] in Iran. It was normalized by Zargar [43]. This scale consists of 41 items, 5 of which constitute the Lie scale. It has a scoring system that ranges from 0 (Completely Disagree) to 3 (Completely Agree). The scoring system will be reversed for items 6, 12,15, and 21. The total score of the questionnaire is calculated by summing all of the items’ scores, except for the Lie scale items. Items 12, 13, 15, 21, and 33 constitute the Lie scale. The scores range from 0 to 108. Higher scores indicate the higher preparedness of the individual responding to addiction. The good psychometric properties of the Iranian version of the IAPS have been confirmed [43].

#### Beck’s depression inventory (BDI-II)

It is a 21-item self-report questionnaire designed to assess the severity of depression and its symptoms. The statements of this questionnaire are rated on a 4-point Likert scale (from zero to three). Higher scores indicate a greater severity of depression [44]. In this test, the minimum score is 0 and the maximum score is 63. Using the test-retest method, the test reliability of the test was obtained in the 0.73–0.93 range. The correlation coefficient between this questionnaire and the Hamilton Depression Rating Scale was 0.6 [44]. This scale has also been widely used in Iran, and its psychometric characteristics have been validated [45].

#### Beck’s suicidal thoughts scale

Beck’s Suicidal Thoughts Scale contains 19 items. On this scale, each item has three options. This questionnaire uses a Likert Scale scoring system with scores ranging from 0 to 2. The total score for the subjects ranges from 0 to 38. In this scale, a score of 0–5 indicates the absence of suicidal ideation, a score of 6–19 indicates preparedness for suicide, and a score of 20–38 indicates an actual suicide attempt. Cronbach’s alpha (internal consistency) and concurrent reliability of this scale were 0.89, 0.83, and 0.96, respectively, displaying a significant correlation with Beck’s Hopelessness Scale and Depression Inventory [46]. According to reports, this scale’s validity and reliability are adequate for Iranian participants [47].

### Statistical analysis

Descriptive statistics were used to analyze the demographics of the participants. Before conducting data analysis, the assumptions of normality and non-linearity were checked using kurtosis and skewness indices, and variance inflation factor (VIF) statistics, respectively. The Pearson correlation coefficient was utilized to determine the relationship between the main variables. Additionally, the SEM method was used to investigate the direct and mediated effects. Also, the fit indices of the model were examined to investigate if all the indices were within the acceptance range of the model. The data were analyzed using SPSS Ver.24 and LISREL.

## Results

### The descriptive data and correlation between the research variables

The present study sample consisted of 152 (47.1%) women and 171 (52.9%) men. Also, the mean and standard deviation of the participants’ ages were 28.83 and 7.33, respectively.

Before conducting data analysis, the assumptions of normality and nonlinearity were examined. Table 1 specifies the curvature and kurtosis values used to investigate the normal distributionof the research variables. Chou, & Bentler [48] found a cut-off point of ± 3 to be suitable for obtaining skewness. Generally, skewness index values exceeding ± 10 can be problematic in multivariable studies [49]. The obtained values for the skewness and kurtosis of the variables indicate the realization of the normality hypothesis. To investigate the non-linearity premise, the VIF and tolerance index were utilized. Since none of the values related to the tolerance index is less than 0.40 and none of the values related to VIF is over 10, the non-linearity premise is assumed to be true. Table 1 presents the central tendency, dispersion indices as well as the correlation of the research variables.

**Table 1:** The descriptive indices and correlation matrix of the research variables

Variables	M	SD	1	2	3	4	5	6	Sk	Ku
Trauma Exposure	65.93	7.82	1						0.57	3.27
Substance Use Tendency	95.76	8.67	-0.09	1					-1.54	7.24
Depression Symptoms	33.82	7.73	**0.21	*0.13	1				-1.23	3.60
Suicidal thoughts	7.09	9.40	0.05	0.008	**0.34	1			1.66	1.18
Peritraumatic Dissociation	27.33	3.94	**0.17	-0.09	0.04	0.01	1		0.54	2.51
Experiential Avoidance	35.59	5.93	**0.23	0.09	0.10	-0.03	**0.20	1	0.09	-0.41

**P < 0.01 *P < 0.05

As shown in Table 1, trauma exposure has a significant direct relationship with the following variables at a significance level of 0.01: PD (0.17), EA (0.23), and depression symptoms (0.21). In addition, EA, and suicidal thoughts have a direct and significant relationship with PD (0.20) and depression symptoms (0.34), respectively. The symptoms of depression have a direct and significant relationship with substance use (0.13) at a significance level of 0.05.

### Evaluation of the hypothesized model using SEM

The investigation yielded the following fit indices for the obtained structural model: (X2/Df = 1/461, CFI and IFI = 0.980, GFI = 0.960, RMSEA = 0.038, SRMR = 0.044). All of the indices are within the fit range of the model, based on these values. Therefore, the structure of the hypothetical model has been approved.

**Fig. 1:**
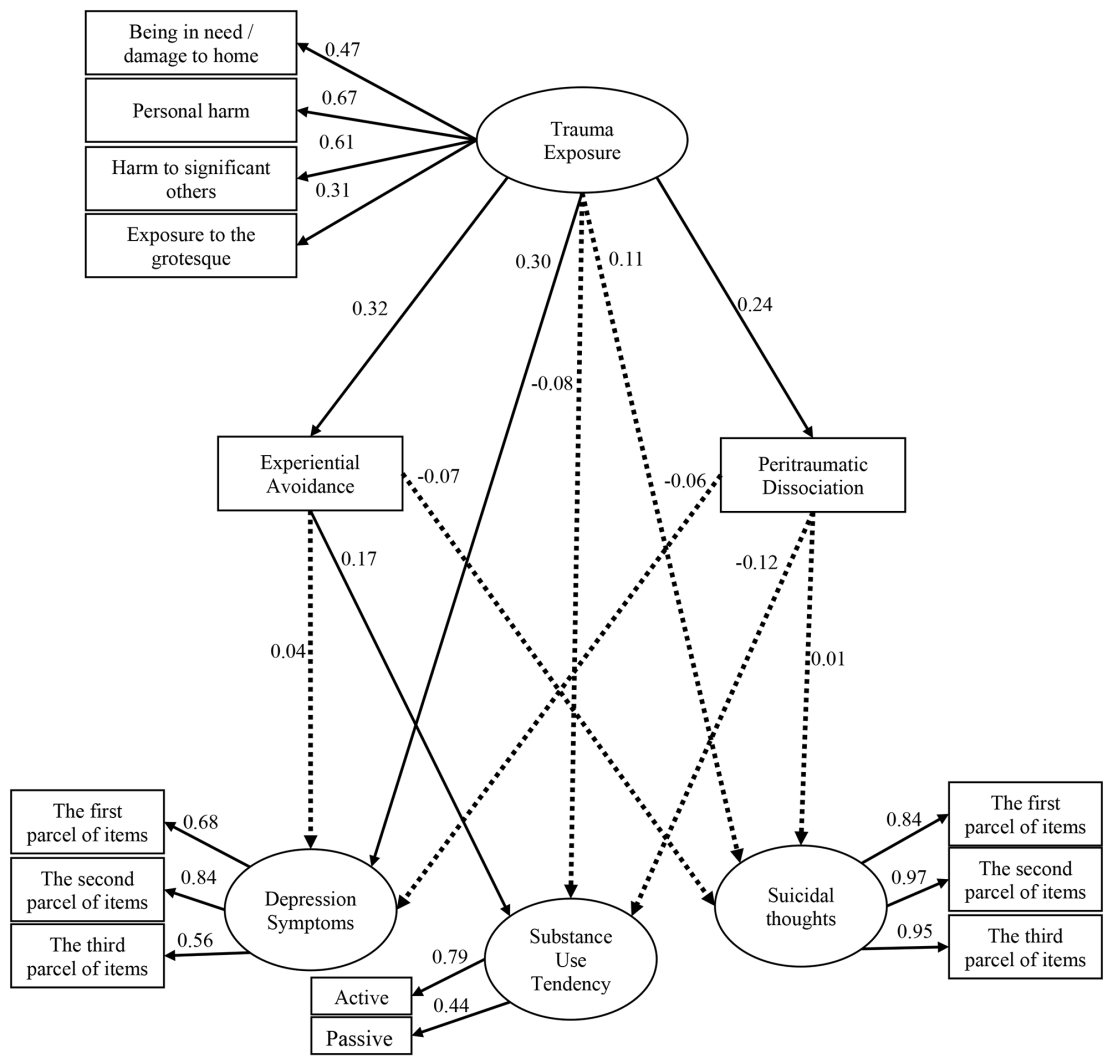
Standard path coefficients of research variables in the main model

To investigate the direct and mediating effects, the structural equation modeling technique was used. The results are represented in Fig. 1 and in tables showing direct and indirect effects. In Fig. 1, significant paths are indicated by solid lines, while non-significant paths are indicated by dashed lines.

### Analysis of direct effects

The direct effect of the research variables is displayed in Table 2. According to Table 2, in cases where the value of the T-statistic is out of range Z (+ 1.96 and − 1.96) or the significant level is less than 0.05, the relationship between the two variables is significant.

**Table 2:** Analysis of direct effects of variables and significance of estimated parameters

Independent variable	Dependent variable	b	β	t	*p*
Trauma Exposure	Depression symptoms	0.34	0.29	3.07	0.002
Experiential Avoidance	Depression symptoms	0.01	0.04	0.65	0.51
Peritraumatic Dissociation	Depression symptoms	-0.03	-0.06	-0.99	0.31
Trauma Exposure	Suicidal thoughts	0.14	0.10	1.35	0.17
Experiential Avoidance	Suicidal thoughts	-0.03	-0.07	-1.14	0.25
Peritraumatic Dissociation	Suicidal thoughts	0.003	0.005	0.09	0.92
Trauma Exposure	Substance Use Tendency	-0.23	-0.08	-0.84	0.39
Experiential Avoidance	Substance Use Tendency	0.15	0.16	2.24	0.02
Peritraumatic Dissociation	Substance Use Tendency	-0.17	-0.12	-1.75	0.08
Trauma Exposure	Experiential Avoidance	0.96	0.31	4.002	0.001
Trauma Exposure	Peritraumatic Dissociation	0.48	0.23	3.17	0.001

It is evident that the direct path from the variable of trauma exposure to the variables of depression symptoms (*T* = 0.298, *β* = 3.079), PD (*T* = 3.178, *β* = 0.236), and EA (*T* = 4.002, *β* = 0.315) is evidently significant. Similarly, the direct path from the variable of EA to the variable of substance use tendency (*T* = 2.241, *β* = 0.166) is also significant. However, the other direct paths among other research variables do not hold significance.

### Analysis of indirect effects

As shown in Table 3, the trauma exposure variable has an indirect and insignificant effect on the depression symptoms (*b* = 0.016, *p* > 0.05) and suicidal thoughts variables (*b* = -0.029, *p* > 0.05) through EA. However the indirect effect of the trauma exposure variable on the tendency to use substances through EA (*b* = 0.152, *p* < 0.05) is significant. Also, the indirect effects of trauma exposure on depression symptoms (*b* = -0.017, *p* > 0.05) substance use tendency (*b* = -0.086, *p* > 0.05), and suicidal thoughts (*b* = 0.002, *p* > 0.05) through PD are not significant.

**Table 3:** Analysis of indirect effects of variables in the research model

Independent variable	Mediator variable	Dependent variable	b	Lower CI	Upper CI	*p*
Trauma Exposure	Experiential Avoidance	Depressive Symptoms	0.01	-0.03	0.09	0.47
Trauma Exposure	Experiential Avoidance	Substance Use Tendency	0.15	0.003	0.44	0.04
Trauma Exposure	Experiential Avoidance	Suicidal thoughts	-0.02	-0.10	0.01	0.25
Trauma Exposure	Peritraumatic Dissociation	Depressive Symptoms	-0.01	-0.10	0.01	0.26
Trauma Exposure	Peritraumatic Dissociation	Substance Use Tendency	-0.08	-0.37	0.007	0.06
Trauma Exposure	Peritraumatic Dissociation	Suicidal thoughts	0.002	-0.04	0.03	0.91

## Discussion

This study aimed to explore the relationships between trauma exposure and substance use tendency, depression, and suicidal thoughts, with the mediating role of PD and EA. The results indicated that the intensity of trauma exposure is directly and significantly linked to symptoms of depression. These results are consistent with those of Bavafa et al. [5], Gao et al. [6] and Ide-Okochi et al. [33]. An interpretation of this finding is that depression occurs after a traumatic even — loss, death of a child, injury, illness of a family member, and losing job and resources are common factors for depression [50]. In other words, natural trauma is in a way correlated with relational trauma. Hence, the loss of loved ones, and the complete destruction of the existing social network, resources, and support will have certain psychological consequences, such as depression. In addition, research evidence shows that the less severe the disaster or traumatic event, the more important pre-disaster variables such as neuroticism or a history of psychiatric disorder appear to be [51]. Besides, the more severe the stressor, the less pre-existing psychiatric disorders predict the outcome. Therefore, the severity of exposure to earthquake trauma in this research, such as damage to the home and property, personal harm, harm to significant others, and exposure to the grotesque can be seen as determinants of depression. These findings suggest the need for developing and implementing policies and interventions to decrease depression symptoms among survivors of natural disasters.

The results also indicated that the intensity of trauma exposure had a significant indirect effect on substance use tendency through EA. The results of this research are consistent with those of Shorey et al. [32] and Shameli and Sadeghzadeh [52]. The avoidance model states that when avoidance is used as a coping strategy to adapt to traumatic events, it can have different shapes, such as aggressive behavior or a tendency to use substances [53]. Poon et al. [54] argue that substance use among young after the earthquake disaster indicates that, in the absence of more adaptive mechanisms, they react to emotional disturbance with recourse to substance, which can be interpreted as a kind of failure in emotion regulation. In this research, EA was represented in the form of a tendency to use substances to avoid negative experiences related to earthquake trauma– agonizing sentiments due to the loss of resources and property, significant others, and exposure to the grotesque. Thus, the severity of exposure to an earthquake alone cannot fully explain the tendency to use substances. In that respect, the emotional reactions of individuals (such as EA) to the consequences of the earthquake are of utmost importance. The findings of this article can help enhance the current understanding of the mechanism behind the tendency to use substances and support clinical interventions that are based on the avoidance model.

Furthermore, the results did not reveal a direct relationship between the severity of trauma exposure and the tendency to use substances and have suicidal thoughts. There are contradictory results regarding an increase or decrease in substance use [4, 55, 56] and suicidal thoughts [7, 57] after trauma experience. Different assumptions have been drawn in this regard. After experiencing trauma, some individuals tend to use drugs to forget the trauma experience, alter their consciousness state, achieve numbness, and disconnect from negative feelings [58]. This is while some react to the losses and changes after the trauma with more positive and adaptive reactions. There are also many hypotheses regarding suicidal and self-harm behavior, including the suppression of dissociative feelings, avoiding acting on suicidal impulses, establishment of interpersonal boundaries, and influencing others through self-punishment [59]. Many factors, such as the severity of the disaster, helping fellow-people, support and social integrity, can explain the increase or decrease in suicide [57, 60]. Therefore, based on the arguments in this research, the intensity of earthquake trauma exposure was not a explain of substance use tendency and suicidal thoughts, suggesting there might be another unexplored mechanism involved.

In addition, the present study also found no evidence of the mediating effect of EA in the relationship between the severity of trauma exposure and depression and suicidal thoughts. The findings of this study do not align with the existing literature [27, 35]. Several factors other than experiential avoidance, such as motivation diversity, personal characteristics, and experiential avoidance measurement, may account for depression and suicidal thoughts. Suicidal thoughts are driven by various motives and are closely linked to emotional distress. These emotions include not only depression but also anxiety, shame, guilt, and other negative emotions [61]. Actually, avoidance is just one of the possible motives for suicide. Brown, Comtois, and Linehan [62] have stated that other factors, such as reducing the burden on others or influencing others. Also, some personal characteristics e.g., resilience, secure attachment, helping others, and mindfulness can function as protective factors to prevent suicide and depression. Apart from EA as one of the factors of depression, other elements can include the function of social factors, attachment style, family relationships, and intimacy. Moreover, EA is an extensive structure understanding whose dimensions on paper is difficult, making it challenging to have a broader and clearer view of this component. EA has also been extensively measured as a generalized trait [63]. Accordingly, further research in the future should be directed toward improving this meta-diagnostic concept and reducing its complexities.

The results also indicated the insignificant indirect effect of the intensity of exposure to trauma on the tendency to use substances, depression, and suicidal thoughts through PD. The findings of this study are not consistent with previous literature. Some studies have highlighted the significance of PD in substance use, depression, and suicidal thoughts [20–22]. Some research has also questioned the role of PD. Dancu et al. [64] stated that PD was not repeated in victims of sexual abuse. The evidence also showed that the relationship between PD and PTSD is nonlinear and correlates with other factors, such as the level of arousal [65]. Marmar et al. [66] reported that dissociation was related to PTSD after controlling factors such as exposure level, distress, locus of control, and social support. Besides, PD may even protect the individual against PTSD and other psychological consequences by limiting the encoding of trauma experiences. These factors may also explain why PD did not mediate the relationship between trauma exposure and depression symptoms in this study.

Moreover, some theorists proposed various classifications of dissociation, such as Continuum–Taxon, State–Trait, Outcome–Mechanism, adaptive-maladaptive [12]. The various methods of categorizing dissociation may have also affected the study outcomes. This study suffers from the classic problem of looking at ‘survivor data,’ meaning that we fail to realize what consequences will befall traumatized individuals without recourse to dissociation [12]. The lack of accurate measures for measuring dissociation, as well as social and cultural expectations regarding it– whether or not it can be a socially acceptable response to trauma– may also have contributed to insignificance of PD in this research. Future researchers should adopt the correlation between dissociation as an unknown component and constructs dissociative in nature to produce and improve a better understanding of the concept of dissociation, thus paving the way for a crisper understanding of the relationship between dissociation and other psychiatric phenomena. Here, unlike EA, PD could not play a significant mediating role. Moreover, the conceptual overlap of these two variables further reinforced our speculations regarding the need for more research and a more accurate assessment of PD.

### Limitations and future research directions

The study samples include individuals who have experienced an earthquake. Thus, generalizing the results to other populations with different types of trauma should be done with proper caution. The study was cross-sectional; therefore, the relationships obtained cannot be interpreted as cause and effect patterns. Measures include self-reporting questionnaires. Moreover, for most participants, the long interval between the occurrence of the disaster and participation in this study (4 years after the earthquake) may influence their retrospective reports of PD. Recollecting dissociative experiences long after facing trauma can produce inaccurate reports of an individual’s actual dissociative behavior. There are also limitations regarding the ability to differentiate pre-traumatic risk factors from post-traumatic reactions. In other words, another limitation of this research is the difficulty of assessing when traumatic events occur and when psychological problems begin.

It is suggested to use more accurate clinical interviews to study PD. In addition, considering the multifaceted reactions related to trauma, the inclusion of both emotional poles (avoidance and emotional expression (can help further explain psychological problems. Future research should use longitudinal designs to examine the temporal dynamics of these variables. Furthermore, even if a model demonstrates that a variable leads to a mediator and subsequently an outcome variable, alternative models might account for the findings more adequately. Other variables (such as anger, shame and guilt, mentalization, and attachment styles) might enhance the understanding of the findings or clarify the connections more effectively.

## Conclusion

In general, there are different susceptible constructs before, during, and after a trauma that can help explain the peritraumatic psychological consequences. In this research, the severity of exposure to trauma was a good explain of depression symptoms, PD and EA. This is while not only the trauma but also how individuals react to it is also of utmost importance. In this study, the severity of trauma exposure could explain the tendency to use substances through EA. Despite the insignificance of PD in this research, a more precise definition and measurement of dissociation and including EA as well as other constructs dissociative in nature can improve our understating of this component and help pave the way and undergird future research on explaining psychological consequences of disasters.

## Data Availability

The datasets used and/or analysed during the current study are available from the corresponding author on reasonable request.
